# The Perivascular Niche and Self-Renewal of Stem Cells

**DOI:** 10.3389/fphys.2015.00367

**Published:** 2015-12-02

**Authors:** Min Oh, Jacques E. Nör

**Affiliations:** ^1^Department of Cariology, Restorative Sciences, Endodontics, University of Michigan School of DentistryAnn Arbor, MI, USA; ^2^Department of Otolaryngology, University of Michigan School of MedicineAnn Arbor, MI, USA; ^3^Department of Biomedical Engineering, University of Michigan College of EngineeringAnn Arbor, MI, USA

**Keywords:** regenerative endodontics, perivascular niche, inflammation, wound healing, tissue engineering

## Abstract

Postnatal stem cells are typically found in niches that provide signaling cues to maintain their self-renewal and multipotency. While stem cell populations may serve distinct purposes within their tissue of origin, understanding the conserved biology of stem cells and their respective niches provides insights to the behavior of these cells during homeostasis and tissue repair. Here, we discuss perivascular niches of two distinct stem cell populations (i.e., hematopoietic stem cells, mesenchymal stem cells) and explore mechanisms that sustain these stem cells postnatally. We highlight work that demonstrates the impact of cellular crosstalk to stem cell self-renewal and maintenance of functional perivascular niches. We also discuss the importance of the crosstalk within the perivascular niche to the biology of stem cells, and describe the regenerative potential of perivascular cells. We postulate that signaling events that establish and/or stabilize the perivascular niche, particularly through the modulation of self-renewing factors, are key to the long-term success of regenerated tissues.

## Introduction

Physiological stem cells enable tissue regeneration and repair. Vacanti and colleagues postulated that knowledge generated through research guided toward the regeneration of living tissues could lead to the cure of certain congenital and hereditary disorders, as well as to the development of strategies for tissue engineering that could address the shortage of donor tissues/organs (Vacanti and Langer, 1999). Successful regeneration of living tissues and/or organs that integrate functionally and properly within the host could also improve the quality of life of patients. Here, we highlight the role of postnatal stem cell populations in tissue repair and regeneration, with focus on their microenvironment (i.e., their niche). Stem cells are maintained in specialized niches, where they are relatively quiescent until external signals (e.g., wound) disrupt this equilibrium and drive their fate through downstream lineages that result in fully differentiated cells. Understanding the biology of these stem cell niches and mechanisms that drive stem cell fate can ultimately provide insights into potential signaling targets that can be exploited to regenerate functional tissues.

### Hematopoietic stem cells

Seminal work led to the identification of hematopoietic progenitor populations that are multipotent and self-renewing (Thomas et al., 1957; Till and McCulloch, 1961; Morrison et al., 1997). Thomas and colleagues introduced the concept of utilizing hematopoietic stem cells (HSC) for regenerative medicine when they infused intravenously suspensions of bone marrow-derived cells into patients that underwent radiation and chemotherapy (Thomas et al., 1957, 1975a). These initial findings led to the development of therapies based on bone marrow transplantation that are now commonly utilized to repopulate lost hematopoietic stem cells (Thomas et al., 1957, 1975a,b). In association with the discovery of HSCs within the bone marrow, these findings contributed significantly to the use of multipotent and self-renewing cell populations for regenerative medicine (Till and McCulloch, 1961). The long-term survival rates of patients, and the existence of donor cells within the bone marrow of long-term survivors, provided compelling evidence that self-renewing cells reside within the bone marrow (Storb et al., 1969; Thomas et al., 1975b). Importantly, evidence of spleen colony-forming cells, derived from stem cell populations, suggested that stem cells reside in “niches” (Becker et al., 1963; Siminovitch et al., 1963; Schofield, 1978).

Several studies exhibited evidence of a perivascular niche for the maintenance of hematopoietic stem cells *in vitro* and *in vivo* (Cardier and Barberá-Guillem, 1997; Ohneda et al., 1998; Li et al., 2004; Kiel et al., 2005; Yao et al., 2005; Ding et al., 2012; Corselli et al., 2013). Kiel and colleagues concluded that hematopoietic stem cells within the spleen and bone marrow were associated with the sinusoidal endothelium, suggesting a perivascular niche (Kiel et al., 2005). Others demonstrated that hematopoietic stem cells localize to heterogeneous vascular niches in the bone marrow including arteries and arterioles, and suggested that these niches regulate their quiescence (Bourke et al., 2009; Kunisaki et al., 2013; Nombela-Arrieta et al., 2013). Indeed, the evidence illustrated that these perivascular niches are comprised of various cell types, each possessing a distinct function to contribute to the maintenance of hematopoietic stem cells. For instance, mesenchymal stromal cells secrete key factors including stem cell factor (SCF) and CXCL12 that contribute to the function of the perivascular niche, and the biology of hematopoietic stem cells (Sugiyama et al., 2006; Méndez-Ferrer et al., 2010; Greenbaum et al., 2013). Notably, emerging evidence suggests that endothelial cell-secreted factors play a critical role in the maintenance of hematopoietic stem cells.

Endothelial cell-secreted factors enabled hematopoietic stem cells to produce a significantly higher number of CFU-S_8_ counts when compared to controls, suggesting that these factors enhance the proliferation and/or survival of the stem cell subpopulation (Li et al., 2004). Conditional knockout mice provided further support to the function of stem cell factor (SCF) to the survival of hematopoietic stem cells. When Ding and colleagues utilized a tamoxifen-inducible conditional knockout system for SCF (*Ubc-creER; Scf*^*fl*∕*fl*^), the hematopoietic stem cell population (CD150^+^CD48^−^Lin^−^Sca1^+^c-Kit^+^) was depleted within the bone marrow and spleen postnatally (Ding et al., 2012). As SCF is a ligand for c-Kit, and inhibiting c-Kit resulted in the loss of hematopoietic progenitor cells, these results suggested that SCF is required for postnatal HSC maintenance (Zsebo et al., 1990; Ogawa et al., 1991; Ding et al., 2012). Furthermore, when they conditionally deleted *Scf* from endothelial cells (*Tie2-Cre; Scf*^*fl*∕−^), the fraction of hematopoietic stem cells decreased significantly (Ding et al., 2012). These observations were confirmed by selective deletion of gp130 expression in endothelial cells, utilizing Cre/*loxP*-mediated recombination (*Tie2-Cre;gp130*^*flox*∕*flox*^) (Yao et al., 2005). Collectively, several studies showed that endothelial cells are necessary for bone marrow homeostasis and regeneration, suggesting that signaling events mediated by endothelial cells play a major role in the maintenance of postnatal hematopoietic stem cells (Hooper et al., 2009; Butler et al., 2010; Kobayashi et al., 2010; Poulos et al., 2013).

### Mesenchymal stem cells

Friedenstein and colleagues discovered non-hematopoietic stem cells adherent to tissue culture conditions capable of forming fibroblastic colony forming units (CFU-F) (Friedenstein et al., 1970). Later coined as “mesenchymal stem cells” (MSC), these cell populations were self-renewing and were capable to give rise to multiple lineages (Caplan, 1991; Prockop, 1997; Pittenger et al., 1999; Bianco, 2007; Sacchetti et al., 2007). However, inconsistencies in defining mesenchymal stem cells presented various challenges to investigators within the field (Dominici et al., 2006). Emerging evidence showed that perivascular cells within the bone marrow exhibited characteristics of mesenchymal stem cells, forming a unique niche (Sacchetti et al., 2007; Méndez-Ferrer et al., 2010).

Perivascular cells were further investigated in various fetal and postnatal human tissues that identified these cell populations as mesenchymal stem cells (da Silva Meirelles et al., 2006; Crisan et al., 2008; Zannettino et al., 2008; Paul et al., 2012). Utilizing flow cell sorting, perivascular cells expressed mesenchymal stem cells markers (e.g., CD10, CD13, CD44, CD73, CD90, CD105) and did not express several markers for other cell types (e.g., CD56, CD106, CD133) (Crisan et al., 2008). Several studies proposed that pericytes exhibit the potential to commit to osteogenic, chondrogenic, and/or adipogenic lineages (Doherty et al., 1998; Farrington-Rock et al., 2004). Notably, long-term cultured perivascular cells possessed the ability to differentiate into mesenchymal stem cell lineages, including chondrocytes, multilocular adipocytes, and osteocytes (da Silva Meirelles et al., 2006; Crisan et al., 2008). Isolated pericytes formed mineralized nodules and structures resembling chondrocytes, and adipocytes both *in vitro* and *in vivo* (Doherty et al., 1998; Farrington-Rock et al., 2004). Furthermore, mRNA analysis of pericytes cultured in inductive conditions showed an upregulation of chondrogenic (i.e., Type II collagen, Sox9, aggrecan) and adipogenic (i.e., peroxisome proliferator-activated receptor gamma [PPAR-γ]) markers (Farrington-Rock et al., 2004). Further investigation into mesenchymal stem cell subpopulations in various tissues led to their identification and characterization within oral tissues, including teeth, periapical structures, and periodontal ligament.

Multipotent and self-renewing subpopulations of MSC-like cells was identified within the dental pulp of permanent (Gronthos et al., 2000) and primary teeth (Miura et al., 2003). Emerging evidence demonstrated that these dental stem cells are capable of differentiating into various other cell types, including osteoblasts (osteocytes), odontoblasts, and adipocytes (Gronthos et al., 2000; Miura et al., 2003). It has been also demonstrated that these cells can differentiate into neural cells (Nosrat et al., 2004; Sakai et al., 2012; De Berdt et al., 2015). Interestingly, these stem cells of dental origin have been implicated in partial recovery of movement when transplanted at spinal cord injury sites in laboratory animals (Sakai et al., 2012; De Berdt et al., 2015). And finally, work from our laboratory has demonstrated that dental pulp stem cells are capable of differentiating into vascular endothelial cells (Cordeiro et al., 2008; Sakai et al., 2010; Bento et al., 2013). Notably, these MSC-derived blood vessels are capable of forming anastomoses with the host vasculature to become functional, i.e., blood-carrying vessels (Cordeiro et al., 2008; Bento et al., 2013).

**Figure 1:**
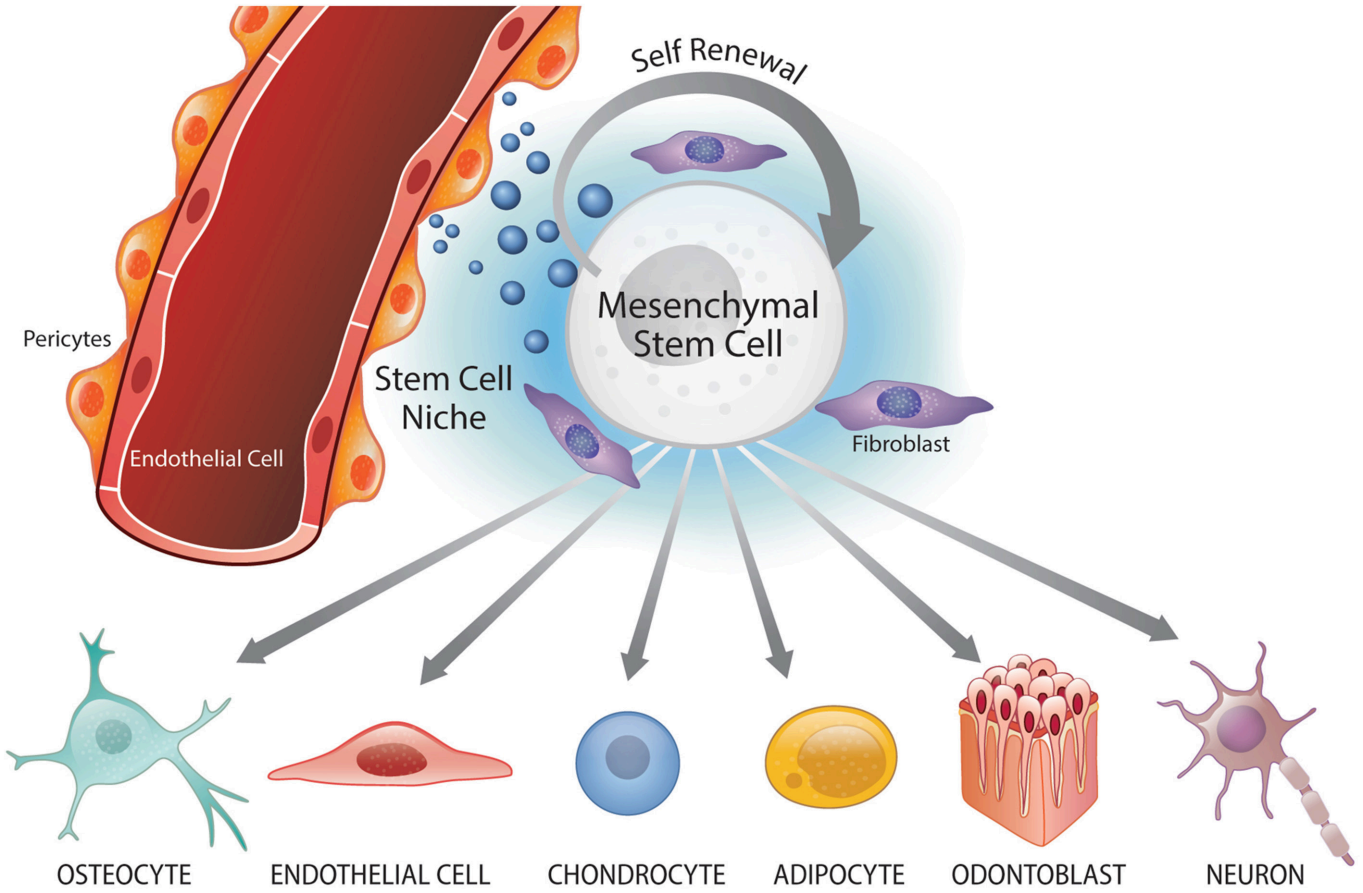
**Perivascular niche and multipotency of mesenchymal stem cells (MSC)**. Mesenchymal stem cells reside in perivascular niches where they undergo self-renewal and maintain the surrounding cells/tissue. Under specific signaling conditions, MSC can undergo differentiation into the osteoblastic (osteocytic), endothelial, chondrocytic, adipocytic, odontoblastic, and neural lineages.

A perivascular niche was identified in postnatal mesenchymal stem cell populations within dental tissues, particularly the dental pulp (Shi and Gronthos, 2003; Machado et al., 2015). These cells residing near the dental pulp blood vessels exhibit hallmark features of stem cells, i.e., multipotency and self-renewal (Figure 1). Seminal work by Shi and colleagues utilized the putative marker STRO1 to identify mesenchymal stem cell subpopulations within the bone marrow and dental pulp, and to verify the potential existence of perivascular niches in these two tissues (Shi and Gronthos, 2003). When STRO1-positive bone marrow stem cells (BMSC) and DPSC were analyzed, they showed expression of pericyte markers (α-smooth muscle actin, CD146) but not von Willebrand factor, a marker for platelets and endothelial cells (Shi and Gronthos, 2003). These findings suggested that these stem cell populations might reside in a perivascular niche and/or have the capacity to differentiate into other cell populations, such as pericytes.

As the origin and behavior of mesenchymal stem cell become better understood, more specific cellular markers can be identified and utilized to identify these cells for investigation or clinical application. Interestingly, recent studies explored potential markers that are more specific to mesenchymal stem cell populations. Lineage tracing studies on Gli1^+^ cells within a murine incisor pulp suggested that Gli1 might be a possible marker for mesenchymal stem cells (Zhao et al., 2014). Utilizing an inducible tagging construct (*Gli1-CE;Zsgreen*) to follow Gli1^+^ cells and their progenitors, ZsGreen^+^ cells were expressed near the cervical loop of mouse incisors within 72 h. After 4-weeks, ZsGreen^+^ cells populated the entire pulp mesenchyme up to the tip of the incisor suggesting that Gli1^+^ cells were responsible for populating the dental pulp. Indeed, ZsGreen^+^ cells were still detected when following Gli1^+^ derivatives for up to 17.5 months, suggesting self-renewal of Gli1^+^ mesenchymal cells (Zhao et al., 2014). Furthermore, Gli1^+^ cells are found in networks around the vasculature, and Gli1^+^PDGFRβ^+^ (platelet derived growth factor receptor-β) cells represent mesenchymal stem cell-like perivascular cells (Zhao et al., 2014; Kramann et al., 2015). The surface marker profile of these mesenchymal stem cell-like perivascular cells was maintained both in culture and *in vivo* (Kramann et al., 2015). Interestingly, as murine incisors develop continuously, dentinal development was severely stunted after vascular damage to the dental pulp, suggesting the functional relevance of the perivascular niche (Zhao et al., 2014). These findings further suggested that mesenchymal stem cell are located and maintained near the host vasculature.

Recent evidence further strengthened the hypothesis that DPSC reside in close proximity to blood vessels and nerves in the dental pulp tissue. These observations were derived from the identification of cells expressing the putative stem cell markers aldehyde dehydrogenase (ALDH)-1, CD90, and STRO1 in close proximity to pulp blood vessels (Machado et al., 2015). DPSC can generate spheroid bodies when cultured in ultra-low attachment conditions, suggesting the existence of a self-renewing subpopulation of cells within DPSC (Xiao et al., 2014). These self-renewing cells form and sustain growth of spheroid bodies in low attachment culture systems (Reynolds and Weiss, 1996; Weiss et al., 1996), particularly in presence of endothelial cell-derived factors. Collectively, these data demonstrated that endothelial cells serve as a source of factors that stimulate self-renewal of mesenchymal stem cells in the dental pulp, thus playing a critical role on the maintenance of the mesenchymal stem cell pool within perivascular niches.

### Stem cell niches and dental tissue regeneration

While the presence of differentiated cells is critical for the function of tissues that have been regenerated, the ability to reconstitute the microenvironment that sustains stem cells is likely important for the successful long-term outcome of the tissue. In fact, it is possible that creation (or regeneration) of the stem cell niche might be sufficient for effective tissue regeneration. Targeting the perivascular niche via regeneration of the vasculature exhibited promising results in the context of dental pulp tissue engineering. A dentin/pulp-like complex was regenerated *in vivo* utilizing a tooth slice/scaffold model of dental pulp regeneration, where tooth slice/scaffolds from human third molars were seeded with SHED (stem cells from exfoliated deciduous teeth) cells and human dermal microvascular endothelial cells (HDMEC) and co-transplanted into the subcutaneous space of severe combined immunodeficient (SCID) mice (Cordeiro et al., 2008; Sakai et al., 2010; Bento et al., 2013). Interestingly, when SHED cells were treated with recombinant human vascular endothelial growth factor (rhVEGF)_165_ within tooth slice/scaffolds *in vitro*, they showed increased angiogenic potential and strong expression of endothelial cell markers (e.g., VEGFR2, PECAM1) (Sakai et al., 2010). These data suggested that these cells had the potential to differentiate into vascular endothelial cells in addition to the expected differentiation into odontoblasts. We observed that SHED cells have the potential to differentiate into endothelial cells *in vivo*, forming functional blood vessels that anastomized with the host vasculature becoming functional (blood-carrying) vessels (Cordeiro et al., 2008; Sakai et al., 2010; Bento et al., 2013). Such data suggested that a sub-population of the dental pulp stem cells could differentiate into tissue-specific odontoblasts while other sub-population may differentiate into vascular endothelial cells possibly recreating perivascular niches for stem cells in the regenerated tissue.

Recent evidence on the effects of endothelial cell-derived factors on head and neck squamous cell carcinoma (HNSCC) cells provided valuable insights on the cellular crosstalk within the perivascular niche. Endothelial cell-derived epidermal growth factor (EGF) promoted epithelial-mesenchymal transition (EMT) of HNSCC cells, endowing them with cancer stem cell characteristics (Zhang et al., 2014). As EMT has been linked to the acquisition of stem cell properties, it is without surprise that endothelial cell-derived EGF induced self-renewal via upregulation of Bmi-1 expression (Zhang et al., 2014). Interestingly, endothelial cells can also stimulate the self-renewal of neural stem cells (Shen et al., 2004). Neural stem cells co-cultured with endothelial cells exhibited delayed differentiation, shown by expression of neural progenitor markers (Nestin^+^ and LeX^+^) and an enhanced neural productive potential (Shen et al., 2004). These data further highlighted the significance of signaling within the perivascular niche for the biology of stem cells.

As stem cells are both, multipotent and self-renewing, a putative approach for tissue regeneration is based on the targeting of self-renewal factors to induce “stemness” of a sub-population of cells. Emulating the niche via controlled regulation of self-renewal pathways might allow stem cells to continue undergoing some level of asymmetric division, where one daughter cell would remain undifferentiated (i.e., self-renewal) while the other daughter cell would undergo differentiation (i.e., multipotency). Emerging evidence demonstrated the important role of self-renewal factors in dental tissue formation. For example, as Bmi-1 was shown to be a key regulator of neural stem cell self-renewal (Molofsky et al., 2003, 2005; Park et al., 2003) Bmi-1^−∕−^ mice incisors exhibited thinner dentinal and enamel layers (Biehs et al., 2013). These data illustrated that self-renewal is essential for odontogenesis, and suggested that this process is likely very important within the context of dental tissue engineering. We are currently designing experiments that will test this hypothesis.

## Conclusions

In summary, the interaction of stem cells with other cellular components of their niche is critical for self-renewal and the maintenance of the stem cell pool, and for the determination of their differentiation fate via multipotency. Mesenchymal stem cells play a vital role in the long-term maintenance of several tissues (da Silva Meirelles et al., 2006; Crisan et al., 2008). Likewise, emerging evidence suggest that mesenchymal stem cells and their niche are critically important for dental tissue regeneration by providing key molecular cues for the maintenance of diverse stem cells populations. We propose that therapeutic efforts to regenerate the stem cell niche are important for tissue engineering. Thus, studies focused on the understanding of conserved mechanisms regulating the biology of stem cell niches will provide valuable insights on the function and maintenance of stem cells, and may have a positive impact on the development of strategies that enhance the long-term outcomes of regenerated tissues and organs.

### Conflict of interest statement

The authors declare that the research was conducted in the absence of any commercial or financial relationships that could be construed as a potential conflict of interest.
